# Recent Advancements in Technologies to Detect Enterohaemorrhagic *Escherichia coli* Shiga Toxins

**DOI:** 10.4014/jmb.2212.12025

**Published:** 2023-02-27

**Authors:** Jeongtae Kim, Jun Bong Lee, Jaewon Park, Chiwan Koo, Moo-Seung Lee

**Affiliations:** 1 Department of Electronic Engineering, Hanbat National University, Daejeon 34158, Republic of Korea; 2 College of Veterinary Medicine & Institute of Veterinary Science, Kangwon National University, Gangwon 24341, Republic of Korea; 3 Green Manufacturing Research Center, Korea University, Seoul 02841, Republic of Korea; 4 Environmental Diseases Research Center, Korea Research Institute of Bioscience and Biotechnology, Daejeon 34141, Republic of Korea; 5 Department of Biomolecular Science, KRIBB School of Bioscience, Korea University of Science and Technology (UST), Daejeon 34113, Republic of Korea

**Keywords:** Shiga toxins, EHEC Stxs, Hemolytic uremic syndrome, sensor, device

## Abstract

Shiga toxin (Stxs)-producing enterohaemorrhagic *Escherichia coli* (EHEC) and *Shigella dysenteriae* serotype 1 are major causative agents of severe bloody diarrhea (known as hemorrhagic colitis) and hemolytic uremic syndrome (HUS) associated with extraintestinal complications such as acute renal failure and neurologic impairment in infected patients under 9 years of age. Extreme nephrotoxicity of Stxs in HUS patients is associated with severe outcomes, highlighting the need to develop technologies to detect low levels of the toxin in environmental or food samples. Currently, the conventional polymerase chain reaction (PCR) or immunoassay is the most broadly used assay to detect the toxin. However, these assays are laborious, time-consuming, and costly. More recently, numerous studies have described novel, highly sensitive, and portable methods for detecting Stxs from EHEC. To contextualize newly emerging Stxs detection methods, we briefly explain the basic principles of these methods, including lateral flow assays, optical detection, and electrical detection. We subsequently describe existing and newly emerging rapid detection technologies to identify and measure Stxs.

## Introduction

Shiga toxin type 2a (Stx2a), together with Shiga toxin type 1a (Stx1a), are the major types of Shiga toxins (Stxs) and form a family of structurally and functionally related bacterial protein toxins. Stxs are mainly produced by the pathogenic bacteria *Shigella dysenteriae* serotype 1 and by Shigatoxigenic *Escherichia coli* serotypes such as O157:H7, O104:H4, and other enterohaemorrhagic *Escherichia coli* (EHEC). A total of 3222 mass outbreak cases (including 39 deaths) were reported in northern Germany from May through to June in 2011. This outbreak strain was typed as an enteroaggregative Shiga toxin-producing *E. coli* O104:H4, producing extended-spectrum beta-lactamase. The Shiga holotoxin consists of two structural subunits A and B. To promote host ribotoxic stress, the enzymatic N-glycosidase A subunit inhibits protein synthesis through catalytic inactivation of the 60S ribosomal subunit by depurinating single adenine residues of 28s rRNA. After endocytosis, the monomeric A subunit is cleaved into two fragments A1 and A2, by the ‘furin’ protease. A1 is the catalytically active fragment, while A2 is essential for holotoxin assembly with the B subunit, which forms a homopentameric structure that binds to globotriaosylceramide (Gb3) glycolipids on the host cell surface. Stx2a is approximately 400 times more toxic (as quantified by LD50 in mice) than Stx1a. Conversely, however, Stx2a is approximately 10-fold less toxic to Vero cells than Stx1a [1]. Researchers have been puzzled by the differential toxicity of Stxs in vitro and in vivo. Since Stxs exhibit equivalent enzymatic activities per ng of protein in vitro [1, 2], it is unlikely that the A subunit is responsible for the differential toxicity of Stx1a and Stx2a. Instead, multiple studies found that the B subunit of Stxs have different binding capacities for Gb3 in target cells. For example, Stx1a has higher affinity for Gb3 in vitro than Stx2a, which may explain the higher toxicity of Stx1a to Vero cells [3]. In vivo, however, a higher affinity for the secondary targets (cholesterol and phosphatidylcholine) of Stx1a but not Stx2a may prevent binding of Stx1a to Gb3, thus attenuating its cytotoxicity [4, 5]. Taken together, these studies indicate a complex interaction of each Stx with Gb3, which may promote differentiate biological activities of Stx1a and Stx2a.

Detection of Stxs relies heavily on timely and optimized analysis technologies, with high levels of accuracy, reliability, and short assay times. Further, detection methods should be fast and easy to use, even by minimally trained or untrained personnel. Importantly, a crucial factor confounding the reliability of these assays is the subjective nature of the assay outputs. Methods with high levels of specificity allow for easier and more reliable interpretation of results. The currently adopted methods of Shiga toxin-producing *Escherichia coli* (STEC) detection are presented herein in the first four subsections. Approaches based on microbiological, DNA/RNA, nucleic acid sequencing, immunology, and biosensing systems are reviewed. We further discuss the use of bench-scale sensors and commercial systems, in addition to emerging technologies. Rapid and accurate identification of STEC is vital for the preservation of human health. Thus, obtaining an accurate diagnosis upon infection is crucial for proper patient care. Conventional detection methods are costly, laborious, time-consuming poorly sensitive, and require the use of highly toxic substances. To identify a pathogenic organism, microbiological and biochemical identification methods require more than 48 h. For these reasons, the culture-based STEC detection, the enzyme-linked immunosorbent assay (ELISA), and radial flow partitioning liquid chromatography (RFPLA), these methods have not received much attention. Nevertheless, a culture-based analysis will remain indispensable in the near future.

However, those approaches still require laborious, time-consuming, complex, and expensive detection processes, as well as trained personnel. These disadvantages become barriers against applying rapid and on-site detection of STEC and Stxs to prevent the spread of food poisoning. To overcome them, recently developed point-of-care detection methods for STEC and Stxs are presented in sections 2.5 to 2.9. They are based on lateral flow assays, optical sensing, and electrochemical sensing and are combined with each other methods, and even with smartphones to achieve higher sensitivity, more rapidity, and high compactivity. We further discuss the recent trends in integration and combination of technologies, as well as the new approaches for high-sensitive detection. Although some cases still require additional systems for high-sensitive detection, they are small in comparison to the conventional equipment used in currently adopted methods. Thus, lateral flow assays, optical sensing, electrochemical sensing, the combination of those technologies, and smart-phone integrated systems are becoming highly suitable for high-sensitive, rapid, and POC detection of STEC and Stxs.

## Stx Detection Technologies

### Microbiological Culture Method for Isolating STEC

Microbiological culture methods have been routinely used to isolate STEC O157:H7 (referred to as O157 STEC) from clinical specimens. Although culture-independent genomic methods have enabled efficient detection of STEC, culture-based methods remain necessary to define their phenotypic characteristics. General culture methods consist of multiple steps: pre-enrichment, selective enrichment, selective and differential plating, serological confirmation, and biochemical screening. The enrichment steps are essential because they ensure full recovery of target bacteria while minimizing the risk of false negatives. The most important issue with these methods is the use of suitable selective and differential culture media for bacteria. For example, O157 STEC can be easily distinguished from other fecal *E. coli* strains by its inability to ferment sorbitol [6]. Thus, sorbitol MacConkey agar (SMAC) is recommended for the isolation of O157 STEC. In clinical laboratories, O157 STEC are isolated according to the guidelines of the Centers for Disease Control and Prevention (CDC) [7]. Briefly, stool specimens from diarrheal patients are sampled aseptically and immediately placed into transport medium. Specimens are transported to clinical laboratories within 48 h inoculated into 3 ml of modified tryptic soy broth (mTSB) before incubation at 37°C for 24 h. To isolate O157 STEC, this enrichment culture is streaked onto cefixime tellurite-sorbitol MacConkey agar (CT-SMAC) or CHROMagar before incubation at 37°C for 24 h [8, 9]. The addition of cefixime and tellurite inhibits the growth of other sorbitol nonfermenting bacteria [9]. After incubation, colorless colonies on CT-SMAC agar or pink colonies on CHROMagar are selected. Each colony is subsequently transferred onto nonselective agar such as Tryptic Soy Agar (TSA), and plates are incubated at 37°C for 24 h. The resultant colonies are analyzed using an O157 antiserum. Since other enteric Gram-negative species may cross-react with O157 antiserum, these isolates should be confirmed as *E. coli* using standard biochemical tests or commercial automated systems (*e.g.*, API20E or Vitek II Compact system) [10].

These conventional culture methods are reliable and accurate for isolation of O157 STEC but not for isolation of non-O157 STEC. Unlike O157 STEC, non-O157 STEC lack the phenotypic characteristics (*e.g.*, sorbitol fermentation) that distinguish them from other *E. coli* strains. To improve the isolation efficiency of non-O157 STEC, the U.S. Food and Drug Administration (FDA) developed a new culture method [11]. This method combines culture and molecular diagnostic techniques and takes 4–5 days. Briefly, 25 g of each sample are taken aseptically and homogenized with 225 ml of modified buffered peptone water with pyruvate (mBPWp). The homogenized mixture is first incubated at 37°C for 5 h, before the addition of antimicrobial supplements to suppress growth of background flora, followed by a second incubation at 42°C overnight [12, 13]. Polymerase chain reaction (PCR) is then performed to detect *stx* and *uidA* genes in genomic DNA extracted from these enrichment cultures. For the isolation of non-O157 STEC colonies, *stx*-positive, *uidA*-negative samples are streaked onto Levine’s eosin-methylene blue (L-EMB) agar or STEC heart infusion washed blood agar supplemented with mitomycin C (SHIBAM), and plates are incubated at 37°C for 24 h [14]. Colonies growing on L-EMB or hemolytic colonies growing on SHIBAM are subsequently restreaked onto TSA supplemented with yeast extract (TASYE) and incubated at 37°C for 24 h. The resultant non-O157 STEC colonies are confirmed by performing a spot indole test and biochemical screening.

These microbiological culture methods are relatively inexpensive and simple but can be time consuming, since they require more than 4 days to obtain a result [11]. Moreover, this process is very labor intensive as it requires preparation of culture media, inoculation into plates, and colony screening. Low sensitivity due to the diversity of microorganisms in the specimens is another limitation of this method [15]. Since a very low infectious dose (10–100 organisms) of STEC can cause illness as early as 2 days after infection, more rapid and sensitive detection methods are required [16].

### Enzyme-Linked Immunosorbent Assay (ELISA)

Since its first introduction in the United States in 1995, enzyme immunoassays (EIAs) have gradually been employed to detect STEC in clinical laboratories. The primary advantage of EIAs using anti-Stx antibodies is that they can identify all serotypes. In addition, EIAs can detect Stx in a direct sample or enrichment culture, providing results more quickly than culture-based methods. To date, the FDA has approved six EIAs for the detection of Stx in human specimens [17-21]. Among them, two EIAs use commercial detection kits that are based on the sandwich ELISA: The Premier EHEC (Meridian Diagnostics, USA) and ProSpecT Shiga Toxins Microplate Assay (Remel, USA) [19, 20]. These ELISA kits were evaluated for their ability to detect Stxs in pure cultures of STEC [22]. Both the Premier EHEC and ProSpecT kits were able to detect the Stx1c, Stx1d, Stx2c, and Stx2f variants produced by STEC. However, they could not detect the Stx2d and Stx2e variants. The detection limits of each ELIA kit when detecting Stxs in pure cultures of STEC were 10^6^ CFU/ml. In addition, the universal procedure of the sandwich ELISA using anti-Stx antibodies was developed and used to detect all subtypes of Stx produced by STEC in ground beef samples [23].

ELISAs are a powerful method for detecting antigen because of their high specificity and sensitivity. Many variations of this assay have been employed including direct ELISA, indirect ELISA, competitive ELISA, and sandwich ELISA. Since the sandwich ELISA is designed to measure quantities of antigen, it is widely used to detect various toxins. This method uses two antibodies: a capture antibody and a detection antibody [24]. A target antigen is bound between a capture antibody immobilized on the microplate well and a detection antibody labeled with a signal-generating enzyme. Subsequently, a suitable substrate (colorimetric, fluorescent, or luminescent) is added to the well, and the resulting signal is proportional to the quantity of target antigen. The specificity and sensitivity of a sandwich ELISA are directly related to the quality of capture and detection antibodies. High-affinity monoclonal antibodies (mAbs) are therefore recommended for use in sandwich ELISAs. Monoclonal antibodies recognize a single epitope of the antigen and thereby provide more reliable results when compared to polyclonal antibodies. In the sandwich ELISA, capture and detector mAbs recognize distinct epitopes on the target antigen and therefore do not interfere with mutual binding capacity. In addition, antigen is purified from the specimen in the capture phase, resulting in a five-fold increase in sensitivity compared with indirect ELISA. A general experimental workflow for sandwich ELISA is outlined as follows. Briefly, microplate wells are coated with a capture antibody diluted in 0.05 M bicarbonate buffer (pH 9.6) at 4°C overnight. After incubation, the capture antibody solutions are aspirated, and plates are blocked with 5% bovine serum albumin (BSA) prepared in 0.05% phosphate buffered saline with tween-20 (PBS-T) at room temperature for 1 h. Next, diluted samples are added to the microplate wells, allowing binding of target antigen to the well. The microplate is incubated at room temperature for 1 h before washing with 0.05% PBS-T to remove unbound antigen. After washing, a detection antibody labeled with horseradish peroxidase (HRP) is added to the wells and incubated at room temperature for 1 h. After incubation, the plate is washed again to remove unbound antibody-HRP conjugates. Sufficient washing between steps is necessary to reduce the background signal from unbound antibody. Finally, a 3,3',5,5'-tetramethylbenzidine (TMB) is added to wells and converted by the antibody bound HRP to a colorimetric signal [25]. The absorbance of the colorimetric signal is subsequently measured using a spectrophotometer.

Despite the accuracy and sensitivity of sandwich ELISAs, they exhibit the following disadvantages: (i) antibody stability may be hampered by inhibitors in complex specimens; (ii) the limited range of the assay, which may require that samples with a higher concentration must first be diluted; and (iii) the cost of preparing high affinity mAbs. To develop more stable and cost-effective methods for detecting Stx, scientists have developed new assays, comparable to sandwich ELISA, but using a multivalent Gb3 probe instead of a detection antibody [26]. Gb3 is a carbohydrate receptor that binds to the B subunit of Stx [27], which can be applied to detect Stx. Although the interaction between single Stx and Gb3 is weak, this limitation can be overcome by using multivalent Stx and Gb3 molecules to enhance binding affinity. Nanoparticles have been used as multivalent Gb3 probes to optimize the Stx-Gb3 interaction [26, 28]. For example, Gb3 analogs immobilized on gold nanoparticles (AuNP) were successfully used to detect Stx1 from *E. coli* cell lysates [26]. In this assay, a capture mAb that recognizes the A subunit of Stx1 was immobilized in wells of a microtiter plate. If Stx is present in sample, it would bind to the well, exposing the Gb3-binding site of the B subunit. Gb3-conjugated AuNP is subsequently added to the wells, and the presence of Stx1 is visualized by silver enhancement. In this case, AuNP reduces silver (I) ions to metallic silver, which is then deposited on the AuNP nuclei. This visible signal can be measured by a plate scanner. Although this method was not as sensitive as ELISA (>1 μg/ml), it provides a relatively cost-effective and rapid alternative for detecting Stx.

### PCR-Based Toxin Detection

PCR is usually recommended to detect Stx-encoding genes in colonies taken from agar plates, but it can also be used on the enrichment cultures [29]. Real-time PCR is a variation of conventional PCR that enables detection of *stx* genes in many public health laboratories [30, 31]. The universal procedure of the real-time PCR using primers specific to *stx1* and *stx2* genes was developed, and its high sensitivity and specificity were verified [32]. Based on the universal procedure, researchers developed multiplex real-time PCR assays targeting several virulence genes for the detection of O157 STEC and non-O157 STEC [33, 34].

Real-time PCR measures the amount of DNA using a fluorescent signal generated during each amplification cycle, whereas conventional PCR only provides a binary output of DNA amplification. Real-time PCR utilizes the exponential phase of DNA amplification to calculate the initial amount of DNA in the reaction. During exponential phase, the amount of DNA doubles with each amplification cycle and is proportional to the fluorescence signal [35]. Thus, the change in fluorescence over time can be used to measure the amount of DNA. When the strength of fluorescence exceeds the threshold at a given cycle, the signal becomes detectable and distinguished from the background. This cycle is known as the threshold cycle (Ct). The Ct is inversely proportional to the starting quantity of the amplification target. If the starting DNA copy number is high, amplification is measured in earlier cycles, and the Ct value is lower, whereas if the initial copy number is low, amplification is measured in later cycles, and the Ct is higher.

The fluorescence signal generated during Real-time PCR can be determined using multiple detection systems. The most widely used systems for detecting STEC are SYBR Green dye-based assays and TaqMan assays. SYBR Green is a fluorescent DNA binding dye, which intercalates with double-stranded DNA (dsDNA) [36]. As dsDNA accumulates during the amplification cycles, dsDNA bound SYBR Green emits a stronger fluorescent signal compared to unbounded SYBR Green. SYBR Green is the simplest and cheapest method for Real-time PCR but is vulnerable to specificity problems as it nonspecifically binds to DNA and can therefore bind to nontarget amplified DNA. A previous study showed the limitation of SYBR Green Real-time PCR for detection of STEC [37]. Its low specificity was identified by the experimental result that only 11 STEC strains were isolated from 35 *stx*-positive meat samples detected by SYBR Green Real-time PCR. Therefore, additional sequence-specific probes are needed to improve the specificity of Real-time PCR.

TaqMan probes are the most widely used sequence-specific assay for Real-time PCR. They consist of an oligonucleotide tagged with a fluorescent dye at the 5’ end and a quencher dye at the 3’ end. TaqMan assays utilize a phenomenon termed fluorescent resonance energy transfer (FRET) [38]. In FRET, the emission of a fluorescent dye is reduced by the quencher dye when two dyes are in proximity. Before PCR begins, the TaqMan probe does not emit fluorescent signal because the fluorescent and quencher dyes are in proximity, enabling FRET. During PCR, the primers and Taq-Man probe hybridize to their complementary sequences on the DNA during the annealing step. In this case, the TaqMan probe still cannot produce signal because the fluorescent and quencher dyes are separated only by the length of the probe. Taq DNA polymerase subsequently extends the primer upstream, and its 5’ to 3’ exonuclease activity cleaves the probe, releasing the dyes. Once cleavage take place, the fluorescent and quencher dyes are separated, increasing the emission of a fluorescent signal.

Recent studies have utilized TaqMan assays to detect *stx* genes according to guidelines from the ISO/TS 13136:2012 (ISO: International Organization for Standardization) [39, 40]. The universal sequence of primers and probes for detecting *stx1* and *stx2* genes has been described previously. Briefly, the total reaction contains 12.5 μl of 2 × TaqMan Master Mix, 500 nM of each primer, 200 nM of probe, 5 μl of DNA template, and molecular biology grade water in a total 25 μl reaction volume. The standard reaction parameters consist of an initial denaturation step at 95°C for 10 min, followed by 45 cycles of 95°C for 15 s and 60°C for 1 min. In addition, the U.S. Department of Agriculture (USDA) has approved TaqMan assay kits commercialized for detecting Stx in livestock products: Rapid-Finder STEC Detection (Life Technologies, USA). Published studies demonstrated that this kit is highly sensitive for detection of STEC and detects all known variants of *stx1* and *stx2* [41, 42]. These TaqMan assays provide more accurate results when compared to SYBR Green systems but require additional design input and cost for the sequence-specific probes tagged with fluorophore and quencher.

Recently, a new gene amplification method termed loop-mediated isothermal amplification (LAMP) has been developed. Since its first introduction in 2000, LAMP has attracted attention as a rapid, highly specific, and cost-effective method for detecting *stx* genes [43]. The universal procedure of the LAMP for detection of Stx2a gene was developed in 2003 [44]. To date, several LAMP assays for detection of STEC in food samples have been developed. For example, the detection limits of LAMP assays for detection of STEC in ground beef samples were 10^3^ to 10^4^ CFU/g, which were comparable to the result of TaqMan Real-time PCR [45].

In the LAMP reaction, pairs of inner and outer primers are used. The forward inner primer (FIP) is complementary to one strand of the amplification region at the 3’-terminal (F1c) and identical to the inner region of the same strand at the 5’-terminal (F2). A DNA polymerase with strand displacement activity initiates DNA synthesis using FIP (F1c+F2). After this initial step, the forward outer primers (F3) bind to their complementary region and displace the previous synthesized single-strand DNA. Because of the F1c sequence in the FIP, the single-stranded DNA can self-anneal and form a loop structure. This strand than serve as the template for DNA synthesis using backward inner primers (BIP, B1c+B2), and subsequent strand displacement is primed by backward outer primers (B3). This allows the other end of the DNA molecule to form a loop structure, resulting in a dumbbell shape with loops at both ends. In subsequent LAMP cycles, a new inner primer binds to the loop region and displaces the synthesized DNA, yielding the original dumb-bell-like DNA and a new dumbbell-like DNA with a stem twice as long. The cycling reaction continues with accumulation up to 10^9^ DNA copies under isothermal conditions (60–65°C) within 1 h. The final products are dumbbell-like DNAs with various stem lengths that are connected to an inverted repeat structure at the amplified region. Although the complex designing of primers is a major constraint of LAMP, this technique can provide highly specific DNA amplification using simple instruments such as water baths. Several published studies have developed LAMP assays for detecting *stx1* and *stx2* genes in food samples [45, 46]. In addition, a LAMP STEC detection kit (Eiken Chemical Co., Ltd., Japan) is commercially available [47, 48].

Although Real-time PCR and LAMP have been used by many public health laboratories, such DNA-based assays have three major limitations: they (i) cannot detect the expression level of Stx, (ii) cannot differentiate active toxins from inactive toxins, and (iii) are not approved by the FDA for diagnosis of human STEC infection by clinical laboratories [12].

### Vero Cell Cytotoxicity Assay

Mammalian cell cytotoxicity assays are unique in that they can measure the physiological activity of toxin. The cytotoxic effect of Stx on mammalian cells has been well characterized. Stx preferentially damages microvascular endothelial cells present in the human kidney after translocation from the intestine to the bloodstream [49]. The B subunit of Stx binds to its target Gb3 receptor expressed on endothelial cells. Stx-Gb3 complexes are subsequently endocytosed and translocate to the endoplasmic reticulum via retrograde transport [50]. During retrograde transport, the A subunit is cleaved by furin in the Golgi apparatus. In the endoplasmic reticulum, the disulfide bond between A1 and A2 fragments is broken, and the A1 fragment is released into cytoplasm [51]. The A1 fragment removes an adenine residue from the 28S ribosomal RNA of the 60S Ribosome [52]. This N-glycosidase activity inhibits protein synthesis, resulting in cell death [53].

Vero cell lines, derived from the kidney of an African green monkey, are the most widely used mammalian cell line to assess the cytotoxicity of Stx. The cytotoxic effects of Stx on Vero cells were first reported in 1977, and Stx was subsequently named ‘Verotoxin’ [54]. Studies have demonstrated that Vero cells are susceptible to Stx because they express high levels of Gb3 receptor on their surface [55]. These cells therefore represent the gold standard for detection of active Stx by examining their morphology 48–72 h postintoxication. Microscopically, Stx-intoxicated Vero cells appeared round and shriveled with many detached cells floating in the medium [54]. These morphological changes are distinct from those of Vero cells exposed to *E. coli* heat-labile enterotoxin (LT). With LT, Vero cells appeared enlarged, thick-walled, and refractive, and possessed several filamentous tendrils [56]. LT-intoxicated Vero cells recover their normal appearance within 3 days in medium supplemented with 10% serum, whereas Stx-intoxicated Vero cells did not recover. For more rapid screening of Stx, this assay was modified to include detection of lactate dehydrogenase (LDH) as a biomarker for cytotoxicity released from Vero cells at 12–16 h postintoxication [57]. The presence of Stx can be confirmed by addition of neutralizing anti-Stx antibodies, which rescue the cytopathic effects on Vero cells [58]. Although its high accuracy, Vero cell cytotoxicity assays are not routinely used in most clinical laboratories because they include an initial cell culture step, which requires expertise and facilities, and is time-consuming.

To overcome the burden of assay preparation and improve the sensitivity for rapid screening of STEC, a three-dimensional (3D) Vero cell platform was developed [59]. In this platform, Vero cells are grown in a 3D collagen-matrix, which is distinct from traditional 2D cell monolayers. This 3D collagen-matrix mimics the organization and behavior of tissue in vivo and thereby provides optimized conditions for testing Vero cell cytotoxicity [60, 61]. The 3D Vero cell platform can detect STEC with cytotoxicty values ranging 33-79% at 6 h postinfection, which is faster than the traditional 2D culture assay. Detection limit of the 3D Vero cell platform at 6 h postinfection was estimated to be 10^7^ CFU/ml for STEC and approximately 32 ng/ml for Stx. In addition, the 3D Vero cell platform is suitable for the rapid detection of STEC from raw ground beef samples.

Despite progress in the use of novel detection methods, development of more sophisticated technologies is underway. The recent use of optical sensing analysis to detect Stxs represents a novel and accurate method to detect toxin.

### Lateral Flow Immunoassays

Lateral flow assays (LFA) or Lateral flow immunochromatographic assays (LFIA) are a paper-based platform commonly combined with traditional laboratory methodologies for detection of pathogens in various samples. Paper is an attractive medium as it is simple to use, affordable, biodegradable, and easy to produce and modify. Furthermore, LFIA are easy to use and interpret, and there is no need for expensive instrumentation or highly trained personnel. This platform is therefore the more practical, simple, and widely used method for point-of-care detection of pathogens.

**Fig. 1:**
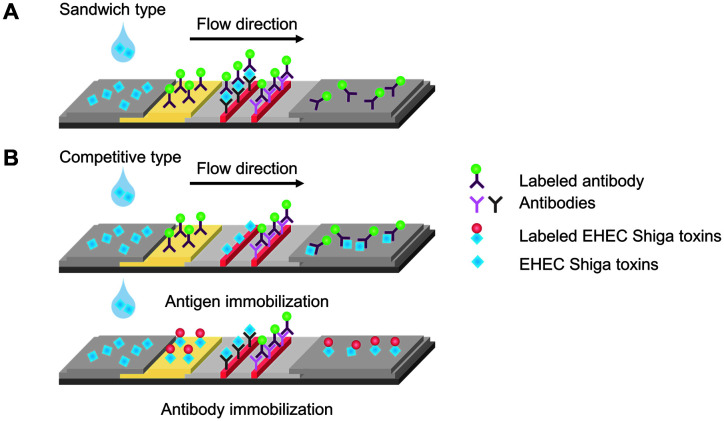
Schematics of lateral flow assays. **A.** Sandwich type. **B.** Competitive type. In (**A**), the antigen-labeled antibody conjugations are captured at detection antibodies at the test zone in the presence of the target antigens, thereby both lines are visible. In (**B**), when the antigens are immobilized at the test zone, the test zone is invisible by target analyte-labeled antibody conjugations. When the antibodies are immobilized at the test zone, it is also invisible by the binding of the target analytes, which are not labeled.

The LFIA device has two standard formats, competitive assays, and sandwich assays, which generate different outputs [62]. These generate continuous flow through the absorbent substrate using capillary action. The sample fluid flows along the substrate, and detectable complexes are formed in certain zones of the test strip (Test line), allowing visualization of assay results as shown in Fig. 1. In the sandwich assay, a positive result shows a visible line in both the test and control zones. In contrast, the competitive assay only shows a visible line in the control zone. Diagnosis results are then inferred by the naked eye.

Despite these benefits, LFIA also have some intrinsic flaws. For example, results discerned by eye are only qualitative, although some reading devices can semiquantify band intensity. Another drawback is the availability of samples in a liquid state, with optimal viscosity to flow through the device, without flow obstruction caused by interfering compounds in the sample. As such, sample pretreatment is required to allow optimal flow through the unit. To mitigate the limitations of LFIA and enhance its performance, various strategies have been explored.

In recent years, major advances in LFIA development have included novel signal-amplification strategies, application of new labels, improved quantification systems, and simultaneous detection. Qi *et al*. first introduced LFIA integrated with competitive and sandwich models to detect multiple targets [63]. In this study, the target analyte was macromolecular (protein). Instead, Wang *et al*. developed a test strip integrated with competitive and sandwich models to detect micro- and macromolecular substances [64]. In this work, they used milk diluted with aflatoxin M1(AFM1) or *E. coli* O157:H7 to validate the LFIA device. This LFIA device can detect AFM1 in the competitive test line and *E. coli* O157:H7 in the sandwich test line. The limits of detections of AFM1 and *E. coli* O157:H7 were 50 pg/ml and 1.58 × 10^4^ CFU/ml, respectively. They also tested real milk samples using their device, but pathogens were not detected as they were present below the LOD of the device.

**Fig. 2:**
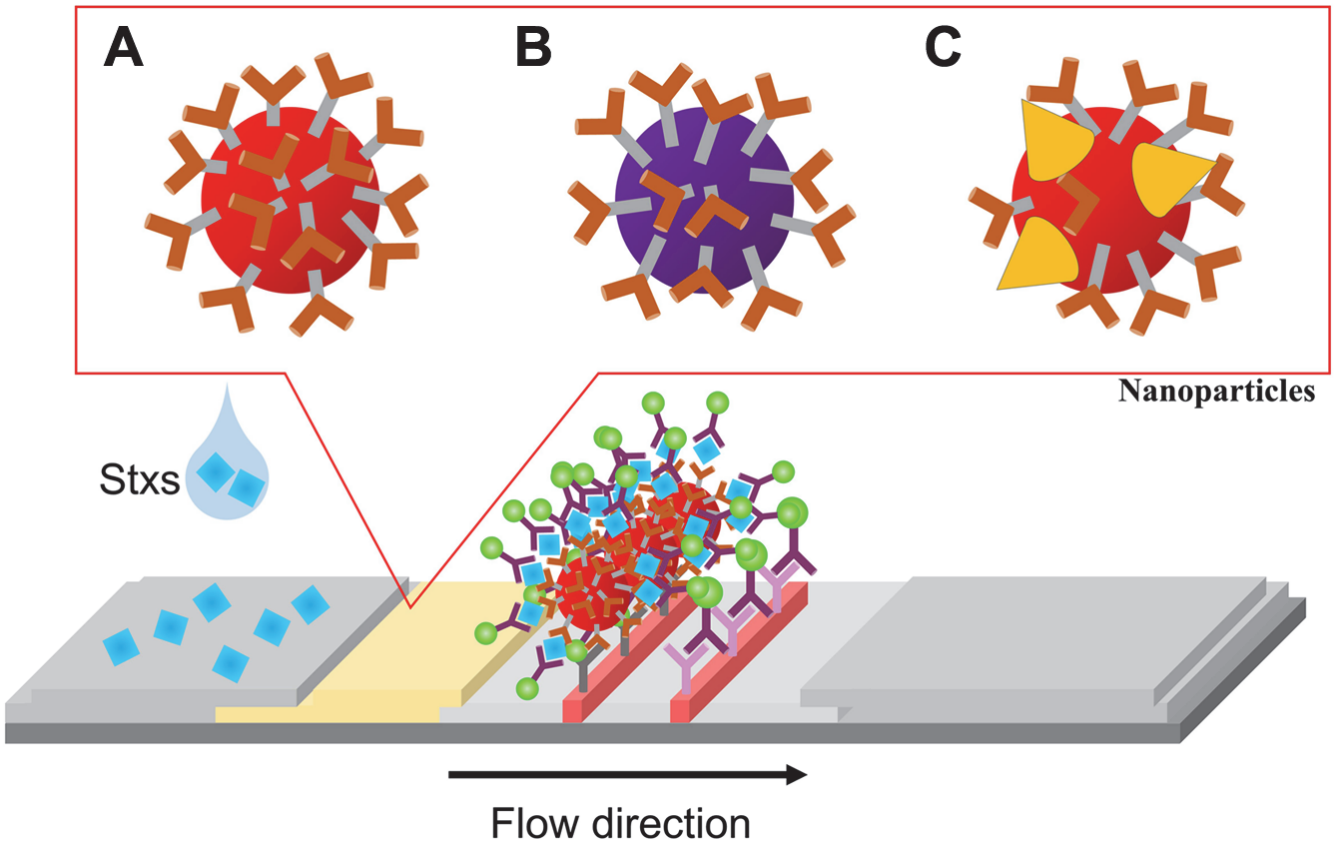
Examples of nanoparticle-based LFA for enhancing sensitivity to EHEC Stxs. (**A**) Colorimetric gold nanoparticle (AuNP). (**B**) Fluorescent quantum dot. (**C**) AuNP with surface-enhanced Raman scattering (AuNP-SERS). In (**C**), AuNPs have nanostructures for Raman scattering.

A novel strategy to enhance the signal is the use of nanoparticles (Fig. 2). Gold nanoparticles have unique optical properties, extraordinary chemical stability, and binding capacity, resulting in their use as a color marker [65]. AuNP-based LFIA are a rapid, simple, and low-cost system for detection of target analytes in various samples with the naked eye [66]. These systems perform well, but low sensitivity problems remain. In addition, the low versatility of the latex nanoparticles inhibits the development of further strategies to combine the strip with transducers. To enhance the sensitivity, the traditional AuNPs label has been replaced by an AuNP nanocomposite and novel nanoparticle, including AuNP-decorated silica nanorods, quantum dots, fluorescence-quenching, and particles used in other emerging detection principles such as magnetic systems [67]. For example, Lu *et al*. reported two chromatographic immunoassays to detect Stx, using the signal la-bels colloidal AuNPs and CdTe QDs, respectively, to reduce the detection limit [68]. The detection limits of the AuNP and QD-based strip are 25 ngml^-1^ and 5 ngml^-1^, respectively. However, the QD-based LFIA system requires additional equipment to excite the QD. Moreover, QD-based LFIA strips have two major drawbacks to overcome. One is that the sensitivity of conventional QD-based LFIA does not enable detection of very low concentrations of toxin. Secondly, large amounts of impurities and inhibitors in food samples may reduce the accuracy of LFIA target detection, generating false negatives [69]. In addition, QDs have some drawbacks, which include water insolubility, toxicity, and lack of functional groups on QDs for bioconjugation [70].

**Fig. 3:**
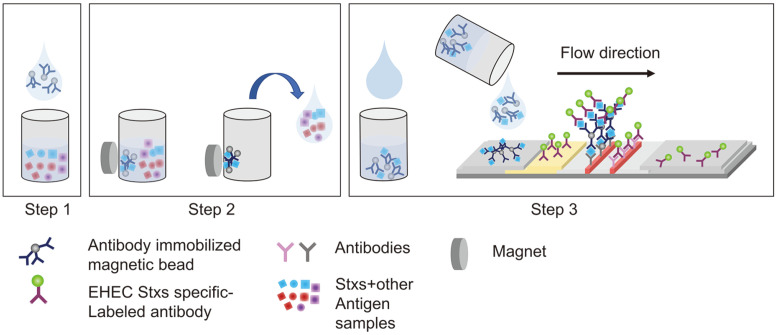
A magnetic immune-separation process and LFA. In step 1, antibody-immobilized magnetic beads were injected into a tube. Using the magnetic field generated by an external magnet, the target antigens were gathered and unbound antigens were removed by a pipette, in step 2. By applying the magnetic beads to the LFA strip, the presence of the target antigens was detected.

Magnetic nanoparticles are essential components of a new generation of biosensors based on LFIA (Fig. 3). MNPs are stable in complex samples and can be manipulated to separate and enrich targets by controlling an external magnetic field [71-73]. They can also be used for quantitative measurements by coupling with an external reader. With these advantages, Lee *et al*. developed an immunomagnetic separation and size-based detection method [74]. They used a test strip that simply overlayed an absorbent pad on an NC membrane with pores. Au-coated magnetic nanoparticle clusters were used to capture *E. coli* O157 in milk. The reported limit of detection of this visual readout for *E. coli* in milk was 10^3^ CFU/ml, which is superior to the typical LFIA (10^5^ CFU/ml).

### Optical Sensing

Optical sensing technologies are a promising method for toxin detection. The main advantages of optical sensing include immunity to electromagnetic interference, durability under severe pressures and temperatures, and high sensitivity mediated by use of unique excitation and emission wavelengths specific to the target analytes.

Fluorescence-based instruments can be categorized into several types based on the parameters they can measure. A fluorometer simply measures fluorescence intensity at a fixed excitation and emission wavelength. Fluorescence lifetime is determined by the decay of emission intensity, which is a unique property of different fluorophores. Anisotropy is the use of a polarized excitation light for characterizing the rotational motions of fluorophores by detection of fluorescence emission with the same polarity as the excitation wavelength.

**Fig. 4:**
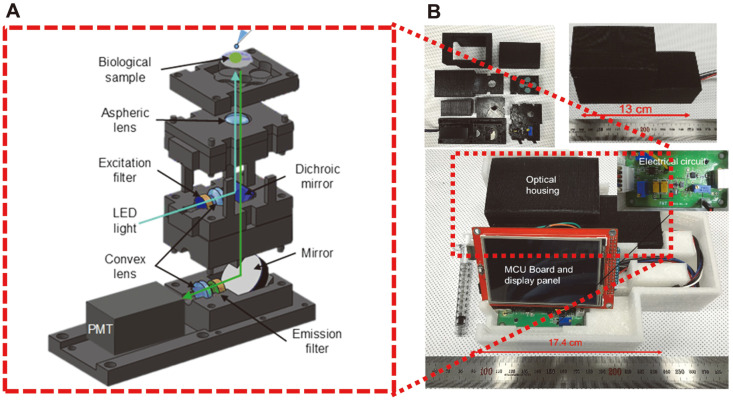
An example of a portable fluorescence detector for sensing EHEC Stxs [63]. (**A**) An optical structure of the fluorescence detector. (**B**) The manufactured portable fluorescence detector.

Filter-based fluorometers are the oldest fluorescence-based detection instruments. As shown in Fig. 4 [75], a target analyte is excited by light passing through a filter to select a higher absorbance wavelength. The excited target analyte emits fluorescence with a longer wavelength than that of the excitation. The emission light, propagated through a beam splitter, is introduced to a photodetector after passing an emission filter to remove light of other wavelengths. Generally, the configuration for a filter-based fluorometer is simpler, cheaper, and smaller compared to a conventional spectrometer due to the absence of conventional monochromators used for excitation and emission. For portable fluorescence detection applications, the filter-based fluorometer is a simple and compact option. However, the optical filters transmit only a single wavelength at a time, limiting assays to a set number of filters for both excitation and emission wavelengths. Therefore, filter-based fluorometers are usually appropriate for applications in which periodic quantitative analysis for a single analyte is needed.

We have reported a highly sensitive and portable (17 × 13 × 9 cm^3^) fluorometer assay for Shiga toxin detection [75]. A 490 nm light emitting diode (LED) was used for excitation, and a commercial photomultiplier tube was used as a photodetector. Although the led has a relatively narrow optical band, it is wider than laser diodes (LD). Thus, an excitation filter was used to exclude surplus wavelengths emitted from the LED. Using this device, we achieved a detection limit of 2 pg/μl when targeting Alexafluor 488 labeled *stx2*. When using THP-1 monocytes, our device could detect fluorescence from the cells at a concentration of 100 ng/ml. While this limit of detection is not at a low level, it can operate in a bright environment because the detection stage can be covered. Fang *et al*. have reported a handheld fluorescence detector for multiple applications [76]. Their device used a PMT photodetector, comparable to our device, but instead used an LD light source. By minimizing the optical elements, such as lenses and mirrors, this device was very compact at 9.1 × 6.2 × 4.1 cm^3^. In this system, fluorescence was detected using a capillary sample vessel. When used as a fluorometer, their device could detect sodium fluorescein solution with a detection limit of 0.42 nM. This system can also be used as a microcontainer and can therefore also be applied to capillary electrophoresis, flow cytometry, and scanning detection. In the capillary electrophoresis mode, an effective separation length of 20 mm can be achieved with an electrical field strength of 1100 V/cm within 7 s using six FITC-labeled amino acids. In flow cytometry, this system has a detection throughput of 240 cells/min. Recently, Jung *et al*. developed a portable luminometer for bioluminescence detection using a SiPM as a photodetector [77]. They validated this device using two different bioluminescent reporters, Pseudomonas fluorescence M3A and an *E. coli* O157:H7 lysogen containing the integrated PhiV10nluc, which were artificially inoculated into ground beef. They reported a lower detection limit of five cells after less than 12 h incubation. Although the sensitivity of SiPM is lower than that of the conventional PMT due to its large active area and large power consumption, it remains a portable and cost-effective electronic circuit. This study demonstrates the possibility of its use as a luminescence detector and an efficient on-site detector.

**Fig. 5:**
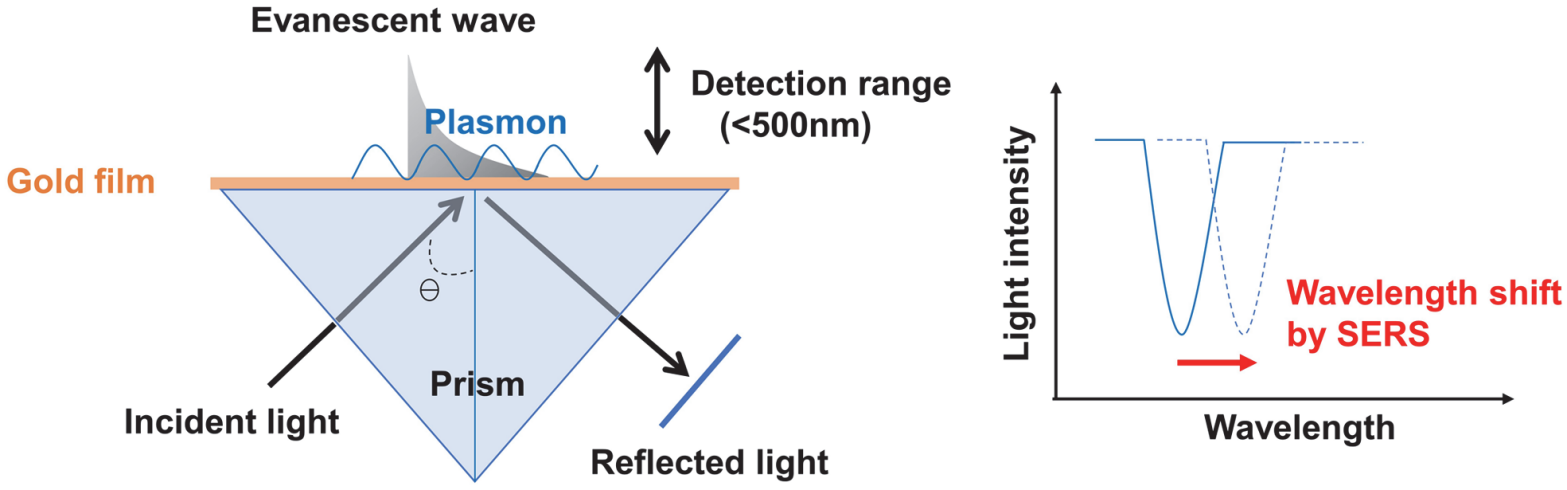
A principle of surface plasmon resonance (SPR) method. When the target antigens are bound to the antibody on the gold film, the wavelength of the reflected light is shifted by Raman scattering.

Recently, many researchers working in biotechnology and clinical chemistry have gained interest in plasmonic nanoparticle-based localized surface plasmon resonance (LSPR) biosensors because of their high sensitivity, low cost, reliability, reproducibility, and selective detection of bacteria [78, 79]. As shown in Fig. 5, LSPR is the resonant oscillation of electrons stimulated by incident light at a metal nanostructured surface covered with a dielectric environment [80, 81]. Its peak wavelength is shifted by the binding of a specific target on the metal surface [78]. LSPR is a well-known label-free detection method, and metal nanostructure-based LSPR sensors have previously been developed and applied extensively in various fields. Recently, various LSPR sensors have been developed and used to detect various bioreactions as well as in immune assays [82-85]. Oh *et al*. developed a simple, portable LSPR-sensing chip incorporating an aptamer and a large area of AuNPs immobilized uniformly on a transparent substrate to monitor *S. typhimurium* contamination [86]. Wang *et al*. developed a label-free, easy-to-use surface plasmon resonance imaging (SPRI) immunosensor capable of high-throughput microarray detection of Stx1 and Stx2 [87]. Multiple antibodies are spotted by programmed microarray. By amplifying the SPRi signal using AuNPs, they were able to detect ~10 pg/ml of the recombinant toxoids Stx1a* and Stx2a* within 20 min.

Emerging technologies are enabling development of flexible microfabrication systems [88, 89]. A promising class of sensors are based on in-fiber microcavities [90], including a microcavity in-line Mach-Zehnder interferometer (μIMZI) [91]. Microcavities with ~10 microscale diameter are present within fiber cladding, which makes up a fiber core. In this structure, the microcavity splits incident light into two beams, where one remains in the fiber core (reference beam), and another propagates through the cavity (sensing beam). The two beams interfere at the far sidewall of the microcavity, and the optical properties are determined mostly by the size, shape, and orientation of the microcavity. Based on this principle, Janik *et al*. developed an optical fiber aptasensor for label-free bacterial detection [92]. The μIMZI sensor was functionalized using APTES and immobilized peptide aptamers on its surface to detect different concentrations of heat-killed *E. coli* O157:H7. The lower limit of detection in real-time measurements reached 10 CFU/ml.

### Electrochemical Sensing

Electrochemical detection methods have a variety of advantages, such as use with turbid media, good sensitivity, low cost, high integration potential, stability, and rapid response [93]. These methods can detect biological analytes by measuring changes in electrical properties, which occur due to interactions between electrodes and samples. Electrochemical detection methods can be classified into three types according to the measured electrical properties: amperometric (current), potentiometric (voltage), and impedimetric (impedance).

The amperometric method can detect target analytes by measuring an electrical current from enzyme-catalyzed redox reactions or bioaffinity reactions at the working electrode. Specific antibodies are immobilized onto the working electrode to capture target analytes. By binding the target analytes, an electrical current can be generated between the working and reference electrodes. This electrical current can be amplified by enzyme-catalyzed redox reactions, which generate an electroactive product. Using the amperometric method, various electrochemical biosensors have been developed for detection of foodborne pathogens.

The basic principle of potentiometric measurements is that electrochemical potential is proportional to the analyte activity. Potentiometric biosensors are composed of a perm-selective layer and a specific bioactive element, such as an enzyme, which is immobilized on the surface of the working electrodes. Enzyme-catalyzed reactions by a specific biorecognition event consume or generate a chemical species near or on the surface of the working electrodes, which can be measured as a potential or voltage.

Impedimetric biosensors are based on electrochemical impedance spectroscopy (EIS). When a sinusoidal voltage signal is applied at various frequencies to a target analyte, the resulting current determines the impedance as a function of the probed frequencies [93, 94]. Antibodies immobilized on the working electrodes capture the target, causing changes in the electrical impedance. The label-free nature of EIS is a major benefit over amperometric and potentiometric sensors.

A rapid enzyme-linked immunomagnetic electrochemical assay was reported with a lower detection limit of 0.90–1.88 CFU in 25 ml of raw milk for *E. coli* O26 [95]. This study used screen-printed carbon electrodes (SPCEs) and paramagnetic beads for multidetection of different bacteria. By using SPCEs and magnetic immunobeads, they eliminated the preprocessing step of the working electrodes and achieved high sensitivity with direct de-tection of E.coli O26. A sandwich-type, bacteriophage-based biosensor was reported for the detection of STEC serogroups in complex food samples with a lower detection limit of 10–10^2^ CFUg^-1^ or ml-1 in less than 1 h [96]. Bacteriophages are excellent alternatives to antibodies for use as biomarkers because they are inexpensive to propagate, highly host-specific, and stable. To enhance sensitivity, specificity, and reliability, the authors used a sandwich-type amperometric assay using bacteriophages as both capture and detection elements for STEC, and achieved an improved lower detection limit.

A highly sensitive, on-chip, DNA-based sensor for detection of the *stx1* gene from STEC was reported with a lower limit of detection of 100 fM in 20 min, using integrated gold microelectrodes (IDEs) on silicon chips [97]. The working IDE was modified with AuNPs and chitosan gold nanocomposites to capture amine-modified probe DNA by a covalent attachment with methylene blue (MB). The electrostatic signal led to accumulation of MD at the DNA-bound IDE, which greatly improved the sensitivity of the assay. An electrochemical detection platform employing aptamer functionalized boron-carbon nanorods with nickel nanoparticles (BC-Ni nanorods) was developed for the detection of STEC strain *E. coli* O157:H7 in water samples [98]. Its limit of detection is as low as 10 CFU with a dynamic range of 10^0^–10^5^ CFU, after minor preprocessing steps for food and fecal samples. BC-Ni nanorods have excellent electrochemical characteristics, such as high conductivity mediated by the thermal substitution of boron in the carbon lattice and faster charge transport by infusion of Ni nanoparticles. The BC-Ni nanorods were modified by anti-*E. coli* O157:H7 specific aptamer and subsequently achieved high sensitivity. An impedimetric DNA dual biosensor was developed for the simultaneous detection of *E. coli* (based on the presence of the *yaiO* gene) and its *f17* fimbriae, with a lower limit of detection of 0.8 fM and 1.0 fM for *yaiO* and *f17* target DNA, respectively [99]. The authors fabricated a thiolated DNA dual-probe sequence on the nanostructured SPCE with gold nanoparticles by a potential pulse-assisted immobilization and were able to detect both target DNA sequences in *E. coli* genomic DNA both before and after digestion.

### Combination of Technologies

Recently, molecular detection modalities have benefited from studies developing new miniaturized and easy-to-use systems for pathogen diagnostics. A variety of nucleic acid amplification methods have been developed for STEC detection. Although digital PCR and Real-time PCR were previously introduced for subtyping STEC, their widespread application was limited by the need for expensive instruments and reagents. Instead, electrophoresis-based multiplexed PCR assays were developed for the recognition of specific pathogenic genes from STEC [100]. However, a limitation of electrophoresis-based methodologies is the difficulty in discriminating between PCR products of similar size with different nucleotide sequences. Furthermore, electrophoresis is laborious and time consuming.

To overcome these drawbacks, various approaches have been employed to detect STEC, *stx1*, and *stx2*. Shan *et al*. established an accurate and rapid method for detecting STEC and *stx1*/*stx2* using asymmetric PCR (asPCR) combined with lateral flow immuno-assays (LFIA) [101]. In this case, the detection target was a single colony from a MacConkey agar plate. Using asPCR with biotinylated *stx1* and digoxin-labeled *stx2* primers, single-stranded DNA (ssDNA) amplicons were labeled with biotin and/or digoxin. These ssDNA amplicons had tags that enabled labeling with carboxylate-modified red polystyrene microspheres (RPMs) by sequential hybridization to distinguish *stx* types. STEC with different Shiga toxin gene types in milk were determined by the appearance of colored test lines on an LFIA strip. As these combined methods focused on distinguishing Shiga toxin gene types, the limit of detection was not reported.

Recently, isothermal amplification methods have been used for point-of-care testing. Considering the advantages of LAMP and filter-based concentration methods, the LAMP-LFIA method was developed [102]. Using a filtration-based pretreatment step, the overall analysis time was effectively reduced, and sensitivity was enhanced by concentrating bacteria beyond detection limit using a microbial enrichment step. When compared with unfiltered controls, the sensitivity of this assay was increased 100-fold. *E. coli* O157:H7 could be detected in beef at concentrations as low as 10^1^ CFU/g within 3 h.

Another recently developed approach is the recombinase polymerase amplification (RPA) method. Although LAMP is fast, accurate, and easy, there are limitations of requiring multiple temperatures (60°C and 80°C) and the use of six primers. Instead, RPA uses a constant, mesophilic temperature (37–42°C) for incubation within 30 min [103]. RPA, therefore, does not require heat cycling but can generate millions of copies of the target gene with one primer with exponential amplification [104]. Therefore, the combination of RPA and LFIA has significant promise for use in the on-site detection of pathogens. For example, Hu *et al*. reported the application of RPA coupled with a dipstick test to detect pathogens in raw milk [105]. Their method includes a RPA reaction in a volume of only 10 μl at 39–42°C for 10 min. The detection limit of RPA-LFIA for *E. coli* O157:H7 genomic DNA of whole bacterial cells was 1 fg and 4.4 CFU/ml with an inoculated raw milk sample. In addition, The RPA-LFIA is highly specific for *E. coli* O157:H7 as the authors observed no cross-reactivity with 19 nontarget foodborne pathogenic bacteria. In another example, Rani *et al*. used *rfbE*, *fliC*, and *stx* gene targets for RPA-LFIA. Test threshold amplification was observed in 5 min at 37–42°C with no false negatives, and this assay could specifically detect amounts of DNA from pure cultures of *E. coli* O157:H7 as low as 100 ag and 1 fg, for the *stx* and *rfbE* genes, respectively [106]. This technology therefore has significant potential for use in on-site, remote, and resource-limited settings.

### Smartphone-Integrated Detection Systems

Rapid development of the integrated circuit industry has led to the widespread use of smartphones as personal and portable communication devices. Smartphones have advanced computational performance and high-resolution graphic capture and processing, and an open-source operating system. These features can be utilized in POC testing systems for use at home and in the clinic. Since smartphones are widely available, their usage in a POC does not incur additional costs. Moreover, their wireless communication capabilities mean they can be connected to other networks. The ubiquitous nature of smartphones and network connections offers unprecedented opportunities for on-site and remote disease diagnostics and management. Particularly, recent advanced technologies, such as microfluidics, 3D printing, and nanotechnology, have been combined with smartphone-based platforms, leading to intelligent, low cost, and effective POC systems for on-site detection applications. These mobile POC systems can be applied to fields ranging from disease screening, diagnostics, and monitoring systems to detect foodborne pathogens and bioterrorism agents. Considering these advantages, a variety of smartphone-based POC systems have subsequently been developed, including a 3D-printed smartphone-based fluorescence imager combined with the classical sandwich ELISA for detection of *E. coli* O157:H7 [107]. This system is composed of an excitation light source, sample chamber, and signal collection system. For portable usage in the field, a long pass filter and focusing lens were used for image collection instead of expensive, complex optical components. These three parts were assembled in a small housing using a 3D printer. The performance of this system was determined using yogurt and egg inoculated with *E. coli* O157:H7. Fluorescence images of the food samples were captured and processed by the smartphone and converted to intensity values, which allows for quantitative analysis. This method had a lower detection limit of 10 CFU/ml in real food samples. Therefore, the smartphone-based fluorescence imager has high sensitivity and was competitive with commercially available microplate reader-based assays. Despite the disadvantages of requiring post-processing, this system has great potential to be used in diagnostics.

Another example is an analysis system for PCR screening using multifunctional modules built into smartphones [108]. Prior to this study, Park *et al*. described a magnetic particle (MP)-based, visual detection method. This colorimetric method is highly sensitive, reproducible, and has a fast reaction time and decreased processing time. Despite these advantages, this method still requires a benchtop centrifuge. To mitigate this problem, a compact centrifuge, powered by a smartphone was integrated with this PCR screening and analysis system. The smartphone camera was used as a light detector in the system and was coupled with a 3D-printed holder. To evaluate its performance, broth and milk were artificially spiked with *E. coli* O157:H7. The low-power rotor in this system could clearly analyze the whole detection range from 10^1^ to 10^6^ CFU/ml, with a lower limit of detection of 10^1^ CFU/ml. This minimized biosensor system combined with a smartphone offers an advanced analysis tool and demonstrates the applicability of low-power electronic devices in a variety of portable biosensor systems.

Recently, an advanced analytical platform integrated with a smartphone for POC DNA testing was introduced using the LAMP method [109]. The integrated smartphone-based genetic analyzer, named i-Gene, contains a film heater for isothermal heating, an optical system for colorimetric monitoring, and a LAMP chip with multiplex pathogen detection. For pathogen detection, an Eriochrome Black T (EBT)-mediated LAMP reaction was adopted for real-time color-based qualitative and quantitative analyses of three pathogenic bacteria. The i-Gene was composed of a controller-embedded heater; a low-cost macrolens; a white LED ring; and a power pack driving the heater for gene amplification, colorimetric detection, and wireless communication. The total size and weight of this system are 4.5 × 6.3 × 6.0 cm^3^ and 60 g, respectively. Briefly, samples are injected into the chip, and the LAMP process was monitored and captured in real-time by the smartphone. The captured data were transferred to the analysis system using Wi-Fi or Bluetooth. The lower detection limit was 101 copies/μl with *E. coli* O157:H7 as the target. This system was highly cost-effective and has excellent specificity, placing it as a candidate, smartphone-based system for quantitative analysis of pathogen burden. Current technologies for detecting STEC or Stxs have been summarized in Table 1.

## Conclusion

LFA are the most practical, simple, and widely used method for on-site detection of Stxs as they are easy to use and interpret, and do not require additional equipment or highly trained lab workers. However, these assays are not quantitative, can be inhibited by viscous liquid samples, and cannot simultaneously detect multiple toxins. To enhance the performance of LFAs, strategies such as combining sandwich type and competitive type systems for multiple target detection, use of gold nanoparticles, and quantum dots for enhancing sensitivity and immunomagnetic separation for increasing concentrations can be used. Optical sensing technologies are a promising method due to their high sensitivity and specificity. Of these technologies, the fluorometer measures fluorescence intensity at a fixed emission wavelengths after excitation. In addition, the fluorometer is suitable for use as a portable detection system because it consists of few lenses, mirrors, and optical filters. However, the limitation of this system is that it can only detect a single wavelength at a time and contains a relatively large photomultiplier tube. To reduce the size of the portable fluorometer, some researchers have removed lenses or developed a silicon photomultiplier (SiPM), with a large active area and large power consumption. An additional optical detection method is surface plasmon resonance (SPR), which is a well-known label-free detection modality. SPR measures a shifted peak wavelength induced by changes in the refractive index caused by a specific target on the metal surface. A new and promising class of sensors is based on in-fiber microcavities, including a microcavity in-line Mach-Zehnder interferometer (μIMZI). Although this system is not highly sensitive, it has the potential to be assembled into a portable system because it uses microcavities on the glass fiber. A highly integrated electrochemical sensor was developed using integrated gold microelectrodes for sensitive detection and SPCEs with paramagnetic beads for multidetection. Additional detection methods utilize synthetic metal electrodes and can achieve 0.8 fM. However, these methods still require huge laboratory equipment, limiting their wide spread use. Recently, researchers have combined the aforementioned technologies to achieve high sensitivity, rapid, and multitarget detection of STEC. LFA is combined with DNA amplification methods, such as asymmetric PCR, loop-mediated amplification, and RPA, subsequently achieving lower limits of detection of 100 pg. Smartphone-integrated systems are an attractive method because of their widespread use in modern society. The multifunctionality of smartphones, such as computing power and the presence of a camera, has led to their combination with LFA. Using the smartphone, the result of an LFA can be quantitative in point-of-care testing. In DNA-based detection, a microscope is required to observe the fluorescence images. As an alternative to microscopes, the camera of a smartphone can be used to capture fluorescence images in point-of-care testing. This review has highlighted multiple EHEC Stx-detection modalities, and indicates that emerging biosensor systems need to be simple, fast, easy to use for the layperson, portable, and highly specific and sensitive.

## Figures and Tables

**Table 1:** Current technologies for detecting *E.coli* Shiga toxins.

No.	Type	Feature	Target analyte	Detection limit	Ref.
1	Culture CDC, CT-SMAC agar, CHROMagar O157	*E. coli* O157:H7	Not reported	[7]
2		FDA, L-EMB agar, SHIBAM agar	Non-O157 STEC	Not reported	[11]
3		USDA, modified rainbow agar, sheep blood agar	Non-O157 STEC	Not reported	[11]
4	ELISA	Premier EHEC, Sandwich ELISA	All STEC	10^6^ CFU/1 ml of bacterial culture, 7 pg/1 ml of Stx1, 15 pg/1 ml of Stx2	[22, 110, 111]
5		ProSpecT STEC, Sandwich ELISA	All STEC	10^6^ CFU/1 ml of bacterial culture	[22, 111]
6		Ridascreen Verotoxin, Sandwich ELISA	All STEC	10^7^ CFU/1 ml of bacterial culture	[22, 111]
7	Reversed passive latex agglutination	VTEC-Screen Seiken	Stx1 and Stx2	25 ng/1 ml of both toxins	[112]
8	Nanoparticle-based platform Gb3-conjugated gold nanoparticle with silver enhancement	Stx1	1 μg/1 ml of Stx1	[26]
9		Gb3-conjugated magnetic nanoparticle with MALDITOF	Stx1	330 pg/1 ml of Stx1	[28]
10	DNA amplification	Real-time PCR, SYBR Green assay, *stx1*, *stx2*, *uidA*	*E. coli* O157:H7	Not reported	[36]
11		RapidFinder STEC Detection, Real-time PCR, TaqMan assay, *stx1*, *stx2*, *eae*	EHEC	8-280 CFU/25 g of meat and vegetable before enrichment	[42]
12		Real-time PCR, TaqMan assay, *stx1*, *stx2*, *eae*_O26_, *eae*_O111_	Non-O157 STEC (O26, O111)	1-10 CFU/1 g of beef and bovine feces before enrichment	[30]
13		Real-time PCR, TaqMan assay, *stx1*, *stx2*, *eae*, *wzx*	EHEC	1-2 CFU/25 g of ground beef before enrichment	[31]
14		Eiken VTEC Detection, LAMP, *stx1*, *stx2*	All STEC	0.3 log_10_CFU/1 g of different foods before enrichment	[47]
15		Mulpiflex PCR	*E. coli* O157 non-O157 STEC STEC virulence genes/*Salmonella*	5-27 CFU/325 g of *E. coli* O157, 9-36 CFU/325 g of non-O157 STEC	[113]
16		Mulpiflex PCR	Bona fide Big Six STEC	4.5 CFU/25 ml of apple juice	[114]
17		LAMP multiplex detection	*E. coli* O157:H7, *Salmonella* spp., *S. aureus*, and *Cochlodinium polykrikoides* (*C. polykrikoides*)	1.7 × 10^2^ CFU/1 ml of milk	[115]
18		LAMP, multiplex detection	*Salmonella* spp., *Staphylococcus aureus*, and *Escherichia coli* O157:H7 in food	3.0 × 10^1^ CFU/sample of Gramnegative bacteria, 3.0 × 10^2^ CFU/sample of Gram-positive bacteria	[116]
19	Vero cell cytotoxicity assay	2D culture, MTT assay, infection time: 24 h	All STEC	1 ng/1 ml of Stx1, 10 ng/1 ml of Stx2	[111]
20		2D culture, LDH release assay, infection time: 6 h	All STEC	10^7^-10^8^ CFU/1 ml of bacterial culture, 1,000 ng/1 ml of Stx	[59]
21		3D culture, LDH release assay, infection time: 6 h	All STEC	10^7^ CFU/1 ml of bacterial culture, 32 ng/1 ml of Stx	[59]
22	Lateral flow Immunoassay	Integrated with competitive and sandwich models	AFM1 *E. coli* O157:H7	50 pg/ml 1.58 × 104 CFU∙/ml	[64]
23		AuNP-based CdTe QD-based	Stx2	25 ng ml−1 5 ng ml−1	[68]
24		Size-based by immunomagnetic separation	*E. coli* O157	10^3^ CFU/ml	[74]
25		Multiplex	*E. coli* O157:H7 S. Typhimurium	2.6 × 10^3^ CFU/1 g of lettuce	[117]
26		Sol-gel-derived silica ink-coated test strips	*E. coli*		[118]
27		Flinders Technology Associates (FTA) cards and glass fibers	*E. coli*		[119]
28	Optical Method	Fluorometer	Stx2	110 pM	[75]
29		Fluorometer-based multiple application	FITC	0.42 nM	[76]
30		Luminescence detection	*E. coli* O157:H7	Not reported	[77]
31		SPRi	Stx1 and Stx2	10~50 pg/ml	[87]
32		μIMZI	*E. coli* O157:H7	10 CFU/ml	[92]
33		Electrochemical method Screen-printed carbon electrodes (SPCEs) and paramagnetic beads	*E. coli* O26	0.90-1.88 CFU in 25 ml	[95]
34		Sandwich-type bacteriophage-based biosensor	STEC serogroups	10-10^2^ CFUg^-1^ or ml^-1^	[96]
35		Integrated gold microelectrodes (IDEs) on silicon chips	stx1 gene	100 aM	[97]
36		Aptamer functionalized BC-Ni nanorods platform	STEC strain *E. coli* O157:H7	10 CFU	[98]
37		Impedimetric DNA dual biosensor for label-free assay	*E. coli* (*yaiO* gene) and virulent f17 fimbriae DNA	0.8 fM and 1.0 fM	[99]
38	Combined method	asPCR with LFIA	Twenty-four strains of STEC	Not reported	[101]
39		LAMP with LFIA	*E. coli* O157:H7	10 CFU/g	[102]
40		RPA coupled with a dipstick	genomic DNA of *E. coli* O157:H7 and *E. coli* bacteria	1 fg and 4.4 CFU/ml	[105]
41		RPA with LFIA	*E. coli* O157:H7	1 fg/(4-5) CFU/ml	[106]
42	Smartphone-integrated detection	Sandwich ELISA combined	*E. coli* O157:H7	10 CFU/ml	[107]
43		MP-based PCR product separation	*E. coli* O157:H7	10 CFU/ml	[108]
44		LAMP reaction	*E. coli* O157:H7	10 copies/μl	[109]
45	Other assays (paper-based)	Paper-based portable culture device	*E. coli*	10 CFU/ml	[120]
46		AuNP-decorated PDMS paper chip	*E. coli*	57 CFU/ml	[121]
47		Litmus paper	*E. coli*	2 × 10^5^ – 4 × 10^4^ CFU/ml	[122]
48	Other assays (microfluidic)	Color-producing compounds deposited on μPAD	L. monocytogenes, *E. coli*, S. enteric	10 CFU/ml	[123]
49		Multichannel paper chip	*E. coli*	10 CFU/ml	[124]
50		AuNP-coated biochips	*E. coli*	50 CFU/ml	[125]
51		Dieletrophoretic microfluidic chip	*E. coli*	300 CFU/ml	[126]
52		Nanoporous alumina membrane	*E. coli*	100 CFU/ml	[127]
